# Altered functional connectivity in first-episode and recurrent depression: A resting-state functional magnetic resonance imaging study

**DOI:** 10.3389/fneur.2022.922207

**Published:** 2022-09-01

**Authors:** Jifei Sun, Zhongming Du, Yue Ma, Limei Chen, Zhi Wang, Chunlei Guo, Yi Luo, Deqiang Gao, Yang Hong, Lei Zhang, Ming Han, Jiudong Cao, Xiaobing Hou, Xue Xiao, Jing Tian, Xue Yu, Jiliang Fang, Yanping Zhao

**Affiliations:** ^1^Guang'anmen Hospital, China Academy of Chinese Medical Sciences, Beijing, China; ^2^Dongzhimen Hospital, Beijing University of Chinese Medicine, Beijing, China; ^3^Beijing First Hospital of Integrated Chinese and Western Medicine, Beijing, China

**Keywords:** first depressive episode, recurrent depressive episode, functional connectivity, default mode network, cognitive control network, affective network

## Abstract

**Background:**

Functional magnetic resonance imaging (fMRI) studies examining differences in the activity of brain networks between the first depressive episode (FDE) and recurrent depressive episode (RDE) are limited. The current study observed and compared the altered functional connectivity (FC) characteristics in the default mode network (DMN), cognitive control network (CCN), and affective network (AN) between the RDE and FDE. In addition, we further investigated the correlation between abnormal FC and clinical symptoms.

**Methods:**

We recruited 32 patients with the RDE, 31 patients with the FDE, and 30 healthy controls (HCs). All subjects underwent resting-state fMRI. The seed-based FC method was used to analyze the abnormal brain networks in the DMN, CCN, and AN among the three groups and further explore the correlation between abnormal FC and clinical symptoms.

**Results:**

One-way analysis of variance showed significant differences the FC in the DMN, CCN, and AN among the three groups in the frontal, parietal, temporal, and precuneus lobes and cerebellum. Compared with the RDE group, the FDE group generally showed reduced FC in the DMN, CCN, and AN. Compared with the HC group, the FDE group showed reduced FC in the DMN, CCN, and AN, while the RDE group showed reduced FC only in the DMN and AN. Moreover, the FC in the left posterior cingulate cortices and the right inferior temporal gyrus in the RDE group were positively correlated with the 17-item Hamilton Rating Scale for Depression (HAMD-17), and the FC in the left dorsolateral prefrontal cortices and the right precuneus in the FDE group were negatively correlated with the HAMD-17.

**Conclusions:**

The RDE and FDE groups showed multiple abnormal brain networks. However, the alterations of abnormal FC were more extensive and intensive in the FDE group.

## Introduction

Major depressive disorder (MDD) is a common clinical psychiatric disorder characterized by significant and persistent depressed mood, decreased interest, cognitive dysfunction, and somatic symptoms (1). According to the ICD-10, MDD can be classified as recurrent depressive disorder (RDE) and first depressive episode (FDE) (2). Epidemiological surveys show that about 80% of MDD patients are at risk of relapse (3). Once patients recover from MDD, they are at high risk of relapse within 5 years, and most MDD patients will have five to nine depressive episodes in their lifetime (4). The risk of relapse in MDD is directly proportional to the number of episodes (5). However, the similarities and differences of neuropathological mechanisms between the RDE and FDE have been less studied.

Although both RDE and FDE are subtypes of MDD, they may exhibit certain differences in clinical symptoms (6). Previous studies have found differences between the RDE and FDE primarily in depressive symptoms, somatic symptoms, and suicidal thoughts (6–8). Another study also showed no significant differences in anxiety between the RDE and FDE groups, but there were differences in memory, which may be related to the poorer executive functioning of RDE (9). In addition, medical costs for the RDE are higher than those for the FDE, and the RDE causes more severe pain in patients (10, 11). Psychiatrists are increasingly recognizing that prevention of MDD recurrence is an important challenge in the treatment of this disease (12). Therefore, distinguishing the neuropathological mechanisms of the RDE and FDE is important for developing new and effective treatment protocols.

In recent years, resting-state functional magnetic resonance imaging (rs-fMRI) has been gradually applied to mental disorders, such as MDD (13–15), bipolar disorder (16), schizophrenia (17), and autism (18). In addition, rs-fMRI has been applied in the study of the neuropathological mechanisms of depression subtypes (19–21). Functional connectivity (FC) is a commonly studied index of rs-fMRI, representing the temporal correlation of neuronal activity patterns in the brain, and is used to study interconnections and interactions between and within brain networks (22, 23). Previous studies have shown that MDD is associated with abnormal brain activity, including abnormal FC between brain regions. Several studies have shown abnormal FC among the default mode network (DMN) (24, 25), cognitive control network (CCN) (26, 27), and affective network (AN) in MDD patients (28, 29). Therefore, the MDD onset may be associated with dysfunction of multiple brain networks, rather than being caused by a single brain network disorder.

MDD has also been studied previously using a combination of several networks, including adolescent depression or TRD (23, 30–34). Most studies have focused on FC abnormalities in the DMN, CCN, and AN in MDD patients (29, 30, 32–34). The functions of emotional, cognitive, behavioral, and transdiagnostic positive and negative valence systems in depressed patients are closely related to the three networks (DMN, CCN, and AN) (29, 35, 36). A previous study performed an FC analysis in MDD patients and found that compared to healthy controls (HCs), the patients showed reduced FC of the CCN in temporal, parietal, and frontal regions and the limbic system, increased FC of the AN in temporal and occipital regions and the cerebellum, and reduced FC of the DMN in the cerebellum and insula (30). However, the FC differences between the RDE and FDE have been less studied.

Sheline et al. (37) found that patients with MDD had increased FC in the bilateral dorsomedial prefrontal cortex with the DMN, CCN, and AN, suggesting that MDD may be associated with simultaneous dysfunction of these three brain networks. Therefore, it is important to study the functional differences among the DMN, CCN, and AN in patients to understand the underlying neuropathological mechanisms of the RDE and FDE.

Previous studies have found reduced FC in the DMN, dorsal attention network, and somatomotor network in the FDE group compared with the recurrent depression (RD) group, suggesting possible FC hypoconnectivity in multiple brain networks in the FDE group (34). In this study, we used a seed-based FC approach to systematically compare FC abnormalities in the DMN, CCN, and AN in RDE patients, FDE patients, and HCs. We further analyzed the correlation between differential brain regions and clinical features. We hypothesized that the FDE patients exhibits more extensive hypoconnectivity in the DMN, CCN, and AN than the RDE patients.

## Materials and methods

### Participants

A total of 63 outpatients with MDD from the Department of Psychosomatic Medicine, Guang'anmen Hospital, China Academy of Chinese Medical Sciences, and the Department of Psychiatry, Beijing First Hospital of Integrated Chinese and Western Medicine, were recruited. All patients met the Diagnostic and Statistical Manual of Mental Disorders, Fifth Edition (DSM-5) criteria for MDD. We used the 17-item Hamilton Rating Scale for Depression (HAMD-17) (38) to assess the severity of depression in all patients and classified the patients into the RDE group (*n* = 32; mean frequency of recurrence, 1.93 [standard deviation = 0.73]) and the FDE group (*n* = 31, 0 recurrence). The severity of the MDD patients recruited was generally mild to severe. The inclusion criteria were as follows: (1) age 18–60 years, (2) HAMD−17 score > 17, and (3) the FDE group all had their first depressive episode prior to enrollment and were not receiving any antidepressant medication. The patients in the RDE group had a previous history of depression, were treated with antidepressants or other antidepressant therapies in remission, had recently relapsed, and had at least a 4-week washout period from antidepressants and other antidepressant therapies before enrollment. We also included 30 sex- and age-matched (22 female and 8 male). HCs based on the same DSM-5 criteria as patients with MDD who (1) were 18 to 60 years old, (2) had an HAMD-17 score <7, (3) were right-handed, and (4) did not have any history of psychiatric disorders in first-degree relatives.

The exclusion criteria for patients and HCs were as follows: (1) severe mental illness or comorbidity with other diseases, such as cardiovascular and cerebrovascular diseases; (2) a history of drug and alcohol abuse; (3) contraindications to MRI, such as presence of a heart pacemaker, permanent metal teeth, or severe claustrophobia; (4) pregnant or lactating status; and (5) bipolar disorder or suicidal ideation.

All patients were required to sign an informed consent form before enrollment. This study was approved by the Ethics Committee of Guang'anmen Hospital, Chinese Academy of Chinese Medical Sciences.

### Scan acquisition

All subjects in this study underwent MRI using a Magnetom Skyra 3.0 T scanner (Siemens, Erlangen, Germany). The subjects generally underwent rs-MRI scans within 5 days of enrollment. Before the scanning procedure, the patients were instructed to remain awake and avoid active thinking. During the scanning process, the patients were required to wear earplugs and noise-canceling headphones, use a hood to immobilize the head, and lie flat on the examination bed. The scanning procedure involved a localizer scan, high-resolution three-dimensional T1-weighted imaging, and BOLD-fMRI.

The scanning parameters were as follows: for three-dimensional T1-weighted imaging, time repetition/time echo = 2,500/2.98 ms, flip angle = 7°, matrix = 64 × 64, field of view = 256 mm × 256 mm, slice thickness = 1 mm, slice number = 48, slices = 192, and scanning time = 6 min 3 s; for BOLD-fMRI, time repetition/time echo = 2,000/30 ms, flip angle = 90°, matrix = 64 × 64, field of view = 240 mm × 240 mm, slice number = 43, slice thickness/spacing = 3.0/1.0 mm, number of obtained volumes = 200, and scanning time = 6 min 40 s.

### Image processing

#### fMRI data preprocessing

The rs-fMRI data were preprocessed using Data Processing Assistant for rs-fMRI (DPARSF) software (DPABI 5.0, http://www.rfmri.org/DPARSF) (39) in MATLAB (Mathworks Inc., Natick, MA, USA), according to the following procedure: (1) conversion of DICOM raw data to a NIFTI format; (2) removal of the first 10 time points to place the data in a stable state; (3) slice timing; (4) realignment of head motion (removal of patients with head movements > 2 mm in any direction and motor rotation > 2°); (5) the resulting aligned image time series for each subject were each co-registered with the corresponding 3D T1-weighted image, and the Diffeomorphic Anatomical Registration Through Exponentiated Lie Algebra (DARTEL) tool was used to normalize the data of all subjects to Montreal Neurological Institute (MNI) space, which was performed using the MNI coordinate space with 3 × 3 × 3 mm; (6) linear detrending in order to reduce the influence of MRI equipment; (7) regression of covariates, including brain white matter signal, cerebrospinal fluid signal, and head movement parameters; (8) the normalized functional images underwent spatial smoothing (Gaussian kernel with 6-mm FWHM); and (9) filtering (0.01–0.08 Hz).

#### Seed-based functional connectivity

In this study, seed point-based FC was used to investigate the differences among the three groups in the DMN, CCN, and AN. These seed point selections were based on previous studies. For each network, a seed region was selected. The posterior cingulate cortices [PCC, (±5, −49, −25)] were used as the seed point of the DMN (40), the dorsolateral prefrontal cortice [DLPFC, (±36, 27, 29)] were used as the seed point of the CCN (37), and the subgenual anterior cingulate cortex [sgACC, (±10, 35, −2)] was used as the seed point of the AN (37). A spherical region of interest (ROI) with a radius of 6 mm was generated with the seed points selected by each network. The mean values of the time series of the ROI were extracted, and a correlation analysis was performed with the mean values of the time series of whole-brain voxels to obtain the functional connectivity map of the brain, which was then transformed into Z values using Fisher's Z-transformation to obtain the intensity values of FC.

### Statistical analyses

#### Clinical data analysis

Clinical data were analyzed using SPSS 23.0 statistical software (IBM Corporation, Somers, NY, USA). One-way analysis of variance was used to compare age and educational levels among the three groups, and the chi-square test was used to compare sex differences. A two-sample *t*-test was used to compare the duration of illness, HAMD-17 scores, and frequency of recurrence between the two patient groups, with a threshold of P < 0.05 (two-tailed) set as indicating statistical significance.

#### fMRI data analysis

##### Within-group-patterns

Imaging data were analyzed using the DPARSF toolbox, and one-way analysis of variance (ANOVA) based on each network seed point (DMN, CCN, and AN) was used to compare whole-brain FC maps among the three groups. Sex, age, educational level, and mean value of framewise displacement (a metric derived from Jenkinson's formula) were used as covariates. The brain areas with FC differences in the three groups were corrected for Gaussian random fields (GRF). The corrected cluster level was set at *P* < 0.05 (two-tailed), and threshold voxel levels of *P* < 0.005 were defined as statistically different.

##### Between-group-differences

We used DPARSF software to extract the mean FC values of abnormal brain regions in each of the three groups and performed *post hoc* between-group two-sample *t*-test analysis in SPSS 23.0 software to show the difference between the two groups (RDE group vs. FDE group, RDE group vs. HC group, FDE group vs. HC group). Using Bonferroni correction, the threshold was statistically significant at *P* < 0.016 (0.05/3).

##### Correlations-with-symptoms

We extracted the mean FC values (after Fisher's r-to-z transformation) of three different brain regions to verify the relationship between altered FC values and clinical symptoms. We performed a Pearson correlation analysis of the clinical scale scores of each group. Significance was set at a statistical threshold of *P* < 0.05 (two-tailed).

## Results

### Characteristics of research samples

Overall, two patients with the RDE and one with the FDE were excluded because of excessive head movement displacement. Therefore, 30 patients with the RDE, 30 patients with the FDE, and 30 HCs met the inclusion criteria. No significant differences among the three groups were found in terms of sex, age, and years of education. The HAMD-17 scores were not significantly different between the RDE and FDE groups, whereas a significant difference was observed in the duration of illness and frequency of recurrence (Table 1).

**Table 1:** Demographic and clinical characteristics of the study participants.

**Variable**	**RDE (*n* = 30)**	**FDE (*n* = 30)**	**HCs (*n* = 30)**	***t(F)/χ*2**	***P*-value**
Sex (M/F)	9/21	7/23	8/22	0.341	0.843^a^
Age (years)	33.06 ± 10.39	35.86 ± 13.42	36.10 ± 13.03	0.560	0.574^b^
Education (years)	13.83 ± 2.74	14.03 ± 2.99	13.83 ± 3.41	0.043	0.958^b^
Duration of illness (months)	25.66 ± 11.45	2.16 ± 0.91	NA	11.205	<0.001^c*^
HAMD-17 score	23.23 ± 3.53	22.96 ± 3.05	NA	0.313	0.756^c^
Frequency of recurrence	1.93 ± 0.73	0	NA	14.316	<0.001^c*^

RDE, recurrent depressive episode; FDE, first depressive episode; HCs, healthy controls; HAMD-17, 17-item Hamilton Rating Scale for Depression; NA, not applicable.

a P-values of sex distribution among the three groups were obtained using the chi-square test.

b P-value from one-way analysis of variance tests.

c P-value from a two-sample t-test.

* Significant difference.

### Differences in FC in the DMN, CCN, and AN among the RDE, FDE, and HC groups

**Figure 1:**
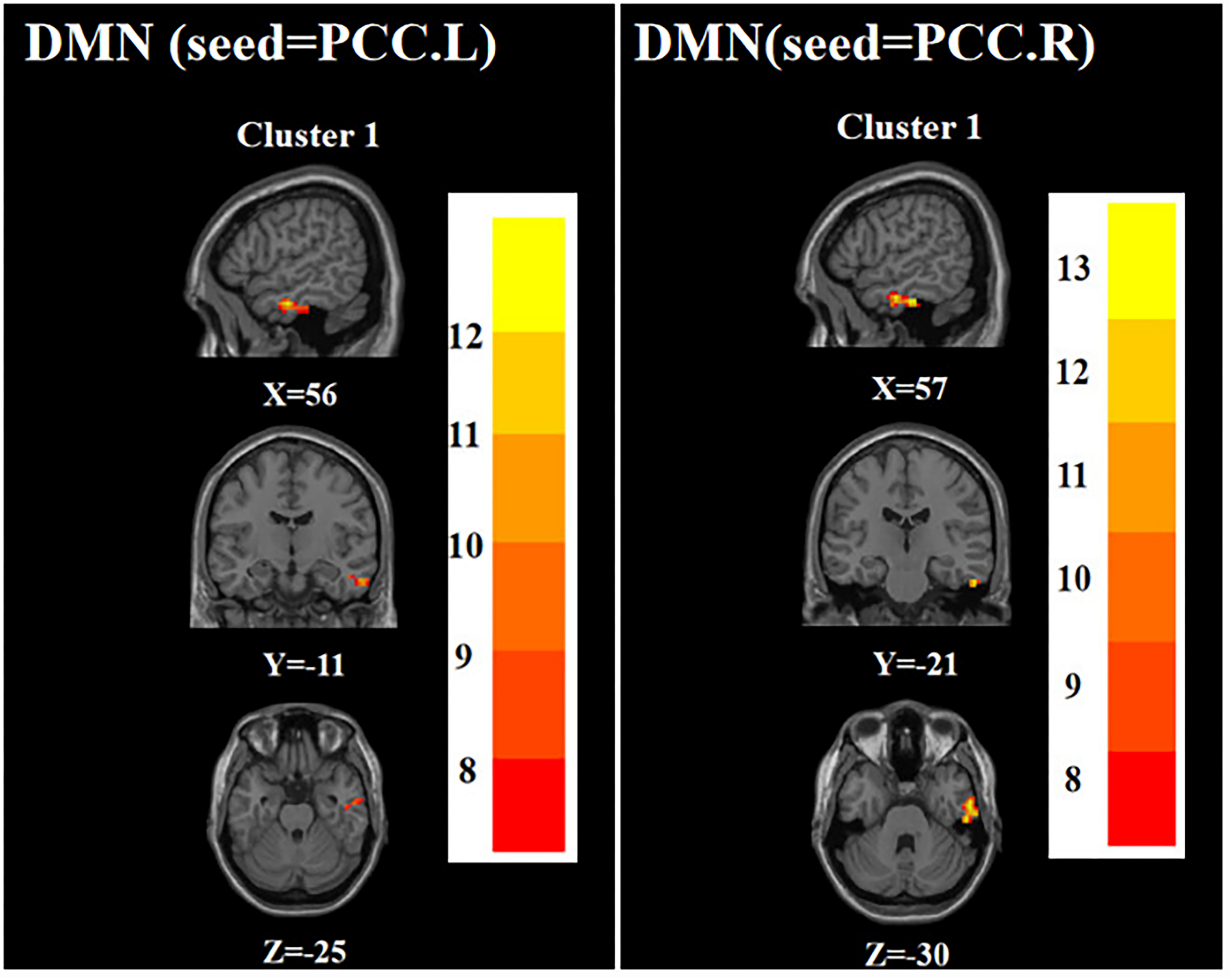
Brain regions with abnormal FC of the DMN among the three groups based on one-way analysis of variance. PCC.L, left posterior cingulate cortex; PCC.R, right posterior cingulate cortex. The color bars indicate the *F*-value.

ANOVA showed that when the left PCC was used as the seed, the right inferior temporal gyrus was significantly different among the three groups. When the right PCC was used as the seed, the right inferior temporal gyrus remained significantly different among the three groups (Table 2; Figure 1).

**Table 2:** FC of the DMN,CCN, and AN differences among the RDE, FDE, and HC groups.

**Cluster**	**Brain regions**	**MNI peak**			**Cluster size**	***F*-value (peak)**
		**X**	**Y**	**Z**		
Differences among three groups of DMN (Seed region: PCC.L)						
1	Right inferior temporal gyrus	56	−11	−25	69	13.071
Differences among three groups of DMN (Seed region: PCC.R)						
1	Right inferior temporal gyrus	57	−21	−30	68	11.570
Differences among three groups of CCN (Seed region: DLPFC.L)						
1	Right precuneus	15	−69	45	48	13.163
Differences among three groups of CCN (Seed region: DLPFC.R)						
1	Left precentral gyrus	−36	4	48	197	9.180
2	Left posterior lobes of the cerebellum	−27	−78	−24	71	10.136
Differences among three groups of AN (Seed region: sgACC.L)						
1	Left hippocampus	−33	−15	−21	67	10.317
2	Left parahippocampal gyrus	−27	−33	−9	44	15.527
Differences among three groups of AN (Seed region: sgACC.R)						
1	Left middle frontal gyrus	−33	63	3	50	10.259
2	Left parahippocampal gyrus	−24	−33	−9	41	12.044

MNI Peak, coordinates of primary peak locations in the Montreal Neurological Institute space.

**Figure 2:**
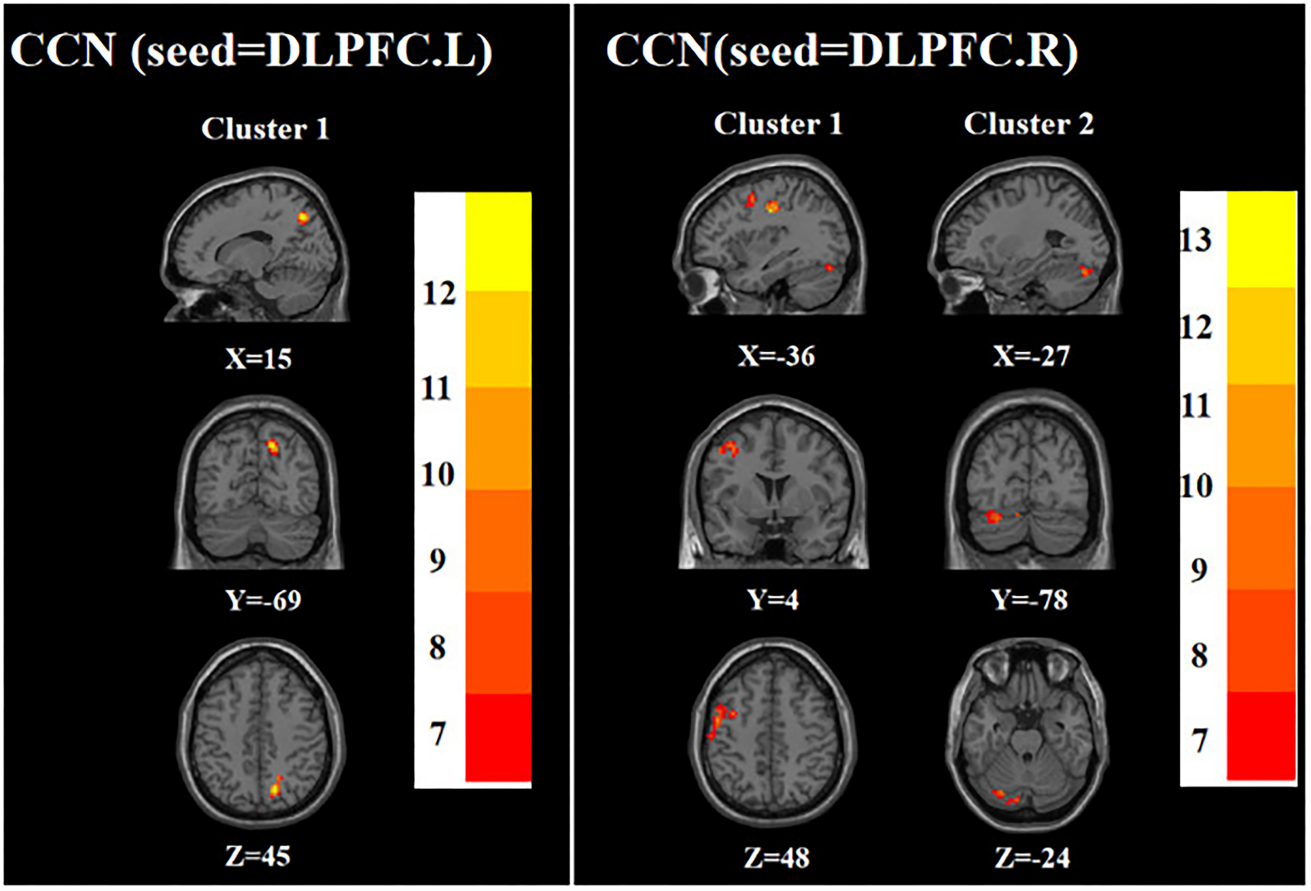
Brain regions with abnormal FC of the CCN among the three groups based on one-way analysis of variance. DLPFC.L, left dorsolateral prefrontal cortex; DLPFC.R, right dorsolateral prefrontal cortex. The color bars indicate the *F*-value.

ANOVA showed that when the left DLPFC was used as the seed, the right precuneus was significantly different among the three groups. When the right DLPFC was used as the seed, the left precentral gyrus and left posterior lobes of the cerebellum were significantly different among the three groups (Table 2; Figure 2).

**Figure 3:**
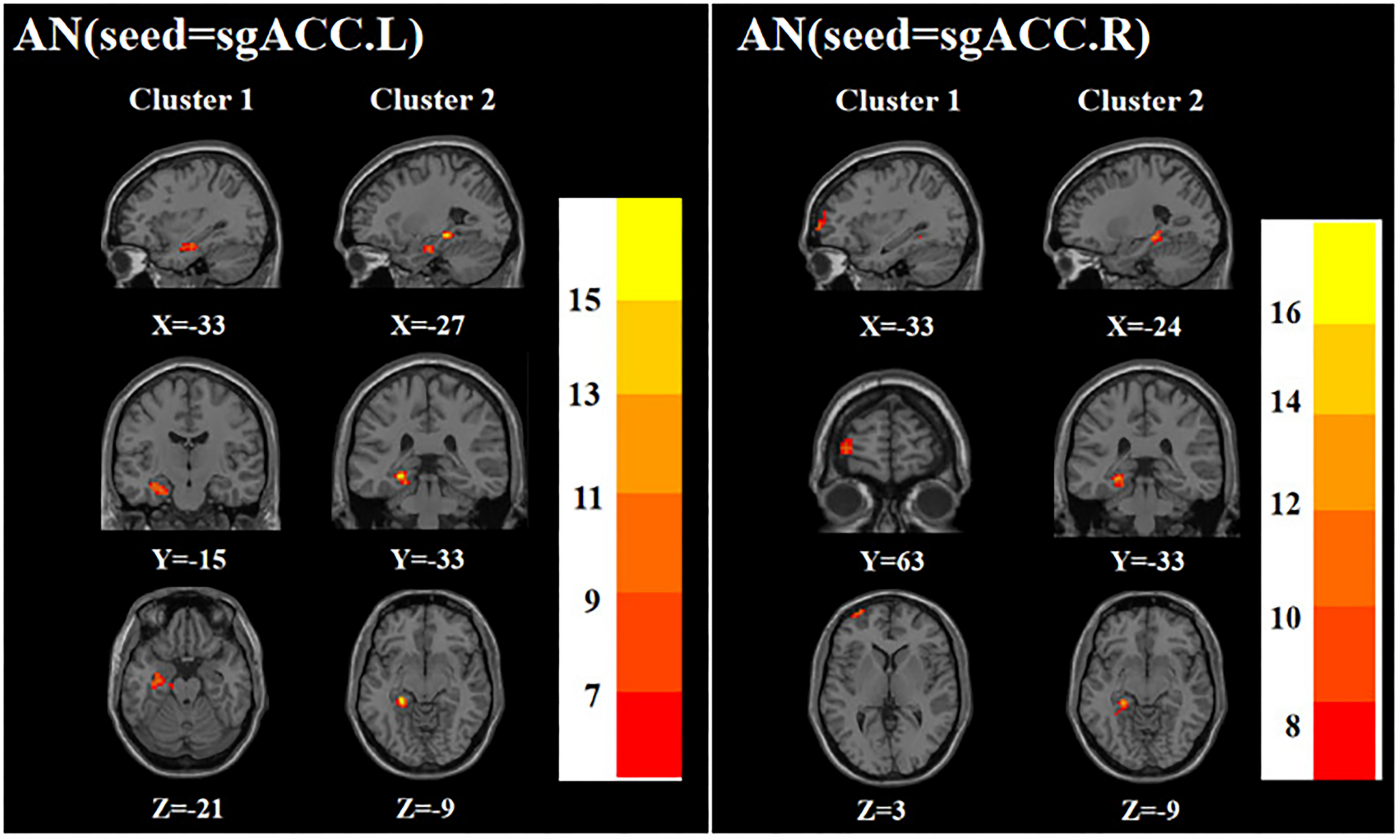
Brain regions with abnormal FC of the AN among the three groups based on one-way analysis of variance. sgACC.L, left subgenual anterior cingulate cortex; sgACC.R, right subgenual anterior cingulate cortex. The color bars indicate the *F*-value.

ANOVA showed that when the left sgACC was used as the seed, the left hippocampus and left parahippocampal gyrus were significantly different among the three groups; when the right sgACC was used as the seed, the left middle frontal gyrus and left parahippocampal gyrus were statistically significantly different among the three groups (Table 2; Figure 3).

### *Post hoc t*-test analysis of differences in FC in the DMN, CCN, and AN among the three groups

**Figure 4:**
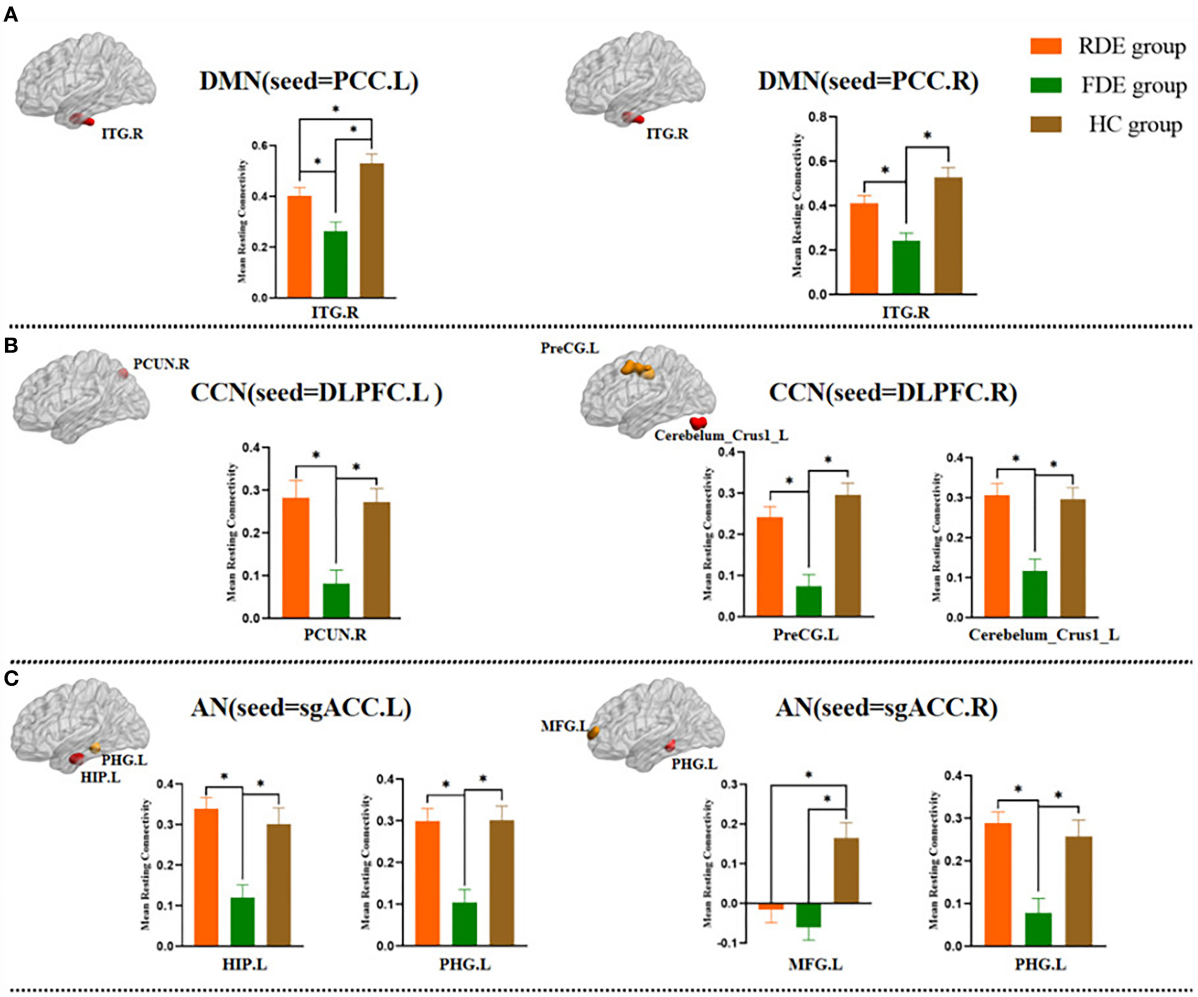
*Post hoc* two-sample *t*-test (Bonferroni corrected) comparison showing FC values differences at peak voxel between each pair group (RDE group vs. FDE group, RDE group vs. HC group, FDE group vs. HC group). ITG, inferior temporal gyrus; PCUN, precuneus; PreCG, precentral gyrus; Cerebelum_Crus1, posterior lobes of the cerebellum; HIP, hippocampus; PHG, parahippocampal gyrus; MFG, middle frontal gyrus. **A**, DMN (seed = PCC); **B**, CCN (seed = DLPFC); **C**, AN (seed = sgACC); L, left; R., right; *P <0.016.

In the DMN, compared with the FDE group, the FC of the left PCC and right PCC with the right inferior temporal gyrus was increased in the RDE group. Compared with the HC group, the FC of the left PCC with the right inferior temporal gyrus was reduced in the RDE group, and the FC of the left PCC and right PCC with the right inferior temporal gyrus was reduced in the FDE group (Figure 4A).

In the CCN, compared with the FDE group, the FC of the left DLPFC with the right precuneus was increased in the RDE group, and the FC of the right DLPFC with the left precentral gyrus, and that in left posterior lobes of the cerebellum were increased in the RDE group. Compared with the HC group, the FC of the left DLPFC with the right precuneus was reduced in the FDE group, and the FC of the right DLPFC with the left precentral gyrus, and that in left posterior lobes of the cerebellum were reduced in the FDE group (Figure 4B).

In the AN, compared with the FDE group, the FC of the left sgACC with the left hippocampus, and that in the left parahippocampal gyrus were increased in the RDE group, and the FC of the right sgACC with the left parahippocampal gyrus was increased in the RDE group. Compared with the HC group, the FC of the right sgACC with the left middle frontal gyrus was reduced in the RDE group, the FC of the left sgACC with the left hippocampus, and that in the left parahippocampal gyrus were reduced in the FDE group, and the FC of the right sgACC with the left middle frontal gyrus, and that in the left parahippocampal gyrus were reduced in the FDE group (Figure 4C).

### Relationship between FC and clinical features

We further performed a Pearson correlation analysis to examine the correlation between FC in abnormally active brain regions and the severity of clinical depressive symptoms.

**Figure 5:**
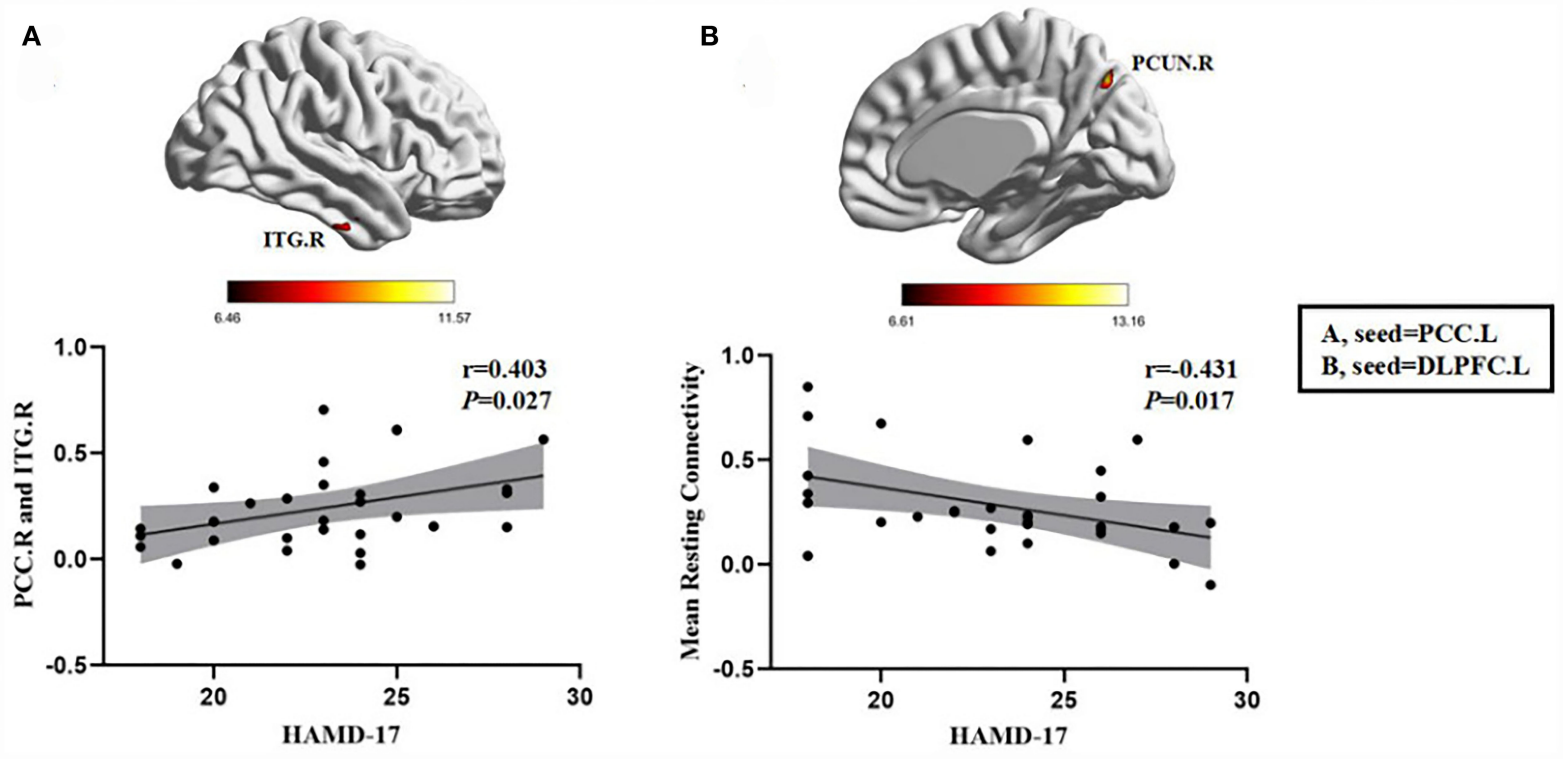
Positive correlation between the FC of abnormal brain regions and the HAMD-17 scores: **(A)** FC values in the FDE group (*n* = 30); negative correlation between the FC values of abnormal brain regions and the HAMD-17 scores: **(B)** FC values in the RDE group (*n* = 30). PCC.L, left posterior cingulate gyrus; DLPFC.L, left dorsolateral prefrontal gyrus; ITG.R, right inferior temporal gyrus; PCUN.R, right precuneus; HAMD-17, 17-item Hamilton Rating Scale for Depression.

In the FDE group, the HAMD-17 scores were positively correlated with the FC between the left PCC and the right inferior temporal gyrus (*r* = 0.403, *P* = 0.027). Moreover, in the RDE group, the HAMD-17 scores were negatively correlated with FC between the left DLPFC and right precuneus (*r* = −0.431, *P* = 0.017) (Figure 5).

## Discussion

This study found multiple abnormal alterations in brain network FC in both the RDE and FDE groups. Compared with the RDE group, the abnormal FC alterations were more extensive and severe in the FDE group. In addition, abnormal changes in the DMN in the FDE group and in the CCN in the RDE group were correlated with disease severity. This study could lead to potential applications for assessing differences in functional brain activity between RDE and FDE.

We found that in the DMN compared to the FDE group, the FC of the left PCC and right PCC with the right inferior temporal gyrus was increased in the RDE group. The functions of the DMN include mainly self-cognitive processes, regulation of emotions, consciousness formation, and memory processing (41–43). Several studies have demonstrated that patients with MDD exhibit functional abnormalities in the DMN and the presence of alterations in the DMN in the onset and remission of depressive symptoms in patients with MDD, hence showing that the DMN plays a very important role in MDD (44–46). The PCC is one of the core regions of the post-DMN, it is closely related to the development of MDD, and may also be linked with the rumination process (47, 48). Studies have shown that intravenous citalopram improves the relationship between DMN connectivity and abnormal serotonin function in patients with MDD and that more ruminative self-evaluation is associated with the FC between the anterior cingulate cortex and the DMN are associated (49). The inferior temporal gyrus is also an important region of the DMN related to spontaneous cognition and verbal fluency that plays an important role in the processing of visual stimuli (50, 51). Meanwhile, in terms of brain structure, MDD patients have diffuse bilateral gray matter loss in the ventrolateral and ventromedial frontal systems, which extends to the temporal gyrus (52, 53). Previous studies have shown that increased FC in DMN is a high-risk factor for the development of MDD (54). Another study showed that the FDE group exhibited reduced FC in the DMN than the RD group, which supports the results of the present study (34). We speculate that the less impaired FC in the RDE group than in the FDE may be related to the protective factors of premedication and the long duration of the disease. Therefore, impaired FC in the PCC and inferior temporal gyrus in the DMN is one of the main differences between the RDE and FDE.

In the CCN, compared with the FDE group, the FC of the left DLPFC with the right precuneus was increased in the RDE group, and the FC of the right DLPFC with the left precentral gyrus, and that of left posterior lobes of the cerebellum were increased in the RDE group. The CCN primarily regulates top–down affective responses and modulation of cognitive and attentional tasks (55, 56). Previous studies have found cognitive dysfunction in MDD patients, and escitalopram can improve DLPFC function and thus alleviate depression and cognitive symptoms (57, 58). The precuneus, located medial to the parietal lobe, is one of the centers of the posterior DMN and is involved in self-related information processing and contextual memory processing (59). A study found the FC of the DLPFC and precuneus was decreased in patients with subthreshold depression compared to HCs (26). Another study also showed a reduced FC between the DLPFC and the angular gyrus in patients with TRD (23). Therefore, the results of this study suggest that abnormal FC between the CCN and post-DMN may be one of the differences in the neuropathological mechanism between the RDE and FDE.

Although the left precentral gyrus belongs to the sensorimotor network (60) and the left posterior lobes of the cerebellum belong to the cerebellar network (61), these structures are also involved in the regulation of the motor system and emotional–cognitive processing (62). Studies have shown that depressed patients have sensorimotor abnormalities that manifest as psychomotor agitation or retardation (63). Previous studies have found FC abnormalities in the DLPFC and precentral gyrus of patients with MDD compared to HCs (64). Another study also found that electroconvulsive therapy-induced improvement of cognitive–emotional impairment in MDD may be related to enhanced FC in the left sgACC and cerebellum (65). Therefore, the results of the present study suggest that differences in the neuropathological mechanisms of the RDE and FDE are also associated with abnormal FC of the CCN with sensorimotor and cerebellar networks, which also explains, to some extent, the complex pathogenesis of RDE.

In the AN, compared with the FDE group, the FC of the left sgACC with the left hippocampus, and that in the left parahippocampal gyrus were increased in the RDE group, and the FC of the right sgACC with the left parahippocampal gyrus was increased in the RDE group. The sgACC is part of the affective network, is involved in emotion processing, mediates motivated behavior, and is part of the AN together with other neural structures, such as the amygdala, hippocampus, and thalamus (66, 67). Previous studies have found FC abnormalities in the left sgACC and left hippocampus in remitted MDD (rMDD) patients compared to HCs (68). Another study also found that the AN in the FDE was reduced compared to FC in the RDE, which is consistent with the present study (34). This suggests that the differences in neuropathological mechanisms between the RDE and FDE are associated with reduced FC of the AN. In addition, the left middle frontal gyrus is located in the DLPFC, an important component of the CCN (64, 69). Studies have shown that the CCN regulates mood by interacting with the sgACC (66). Another study found hyperconnectivities between the sgACC and CCN in rMDD patients compared with HCs, suggesting that these hyperconnectivities may constitute a compensatory mechanism for the increased risk of recurrence (70). Therefore, the results of this study suggest that the abnormal FC of the AN and CCN is one of the differences in the neuropathological mechanisms of the RDE and FDE.

We investigated the correlation between altered brain network functions and clinical depressive symptoms and found that in the CCN of the RDE group, the HAMD-17 scores were negatively correlated with the FC between the left DLPFC and right precuneus. Previous studies found that Beck Depression Inventory scores in the rMDD group were negatively correlated with changes in FC values in the left DLPFC, which is similar to the results of the present study (71). However, we did not find this correlation in the FDE group. This abnormal FC of the CCN-DMN may be an important neuropathological mechanism in the RDE. Previous studies have also shown that transcranial direct current stimulation of the bilateral DLPFC in MDD patients effectively alleviates the risk of recurrence in MDD patients (72). Therefore, the DLPFC may also be a potential therapeutic target for patients with an RDE. On the other hand, we also found a positive correlation between the FC in the left PCC and right inferior temporal gyrus and HAMD-17 scores in the DMN of the FDE group. The correlation between the FC of the PCC and clinical depressive symptoms in FDE patients has been previously reported (73, 74). This also suggests that the onset of an FDE is closely related to impaired DMN function.

Interestingly, we found decreased FC in the DMN, CCN, and AN in the FDE group compared to the HC group, while the RDE group had decreased FC in the DMN and AN only. This suggests that the impairment of the brain network function was more severe in the FDE group, and we speculate that this may be related to the first episode of FDE patients with transient impairment. By contrast, the more modest impairment of brain function in the RDE group may be related to premedication with antidepressants or the course of the disease (75, 76).

Some limitations of this study should be considered. First, in this study, only one seed point in each network (DMN, CCN, and AN) was selected, and the significance level of the analysis was based on the selected seed point as the main concern, which does not fully represent the abnormal functional activity of the whole-brain network. Second, although the RDE patients in this study had discontinued medication 4 weeks before enrollment, there may still be potential antidepressant factors. Third, the number of relapses in RDE patients is inconsistent, and it seems more clinically valuable to compare the results of the first relapse of patients in the RDE group with the results of the FDE group. Finally, the results obtained included clusters with a relatively low number of voxels, which were associated with weak correction thresholds (*P* < 0.005). In addition, no correction was made for the correlation of clinical symptoms with abnormal brain areas. In future studies, we will expand the sample size based on an outpatient population in combination with advertising recruitment methods (77), a more stringent threshold correction (*P* < 0.001), and more scales such as anxiety and rumination to improve the scientific validity of the results of this study.

## Conclusion

The RDE and FDE groups in this study showed multiple abnormal brain network FC alterations in this study. However, the alterations of abnormal FC featured more extensively and intensely in the FDE group.

## Data availability statement

The raw data supporting the conclusions of this article will be made available by the authors, without undue reservation.

## Ethics statement

The experimental protocol was approved by the Ethics Committee of Guang'anmen Hospital, China Academy of Chinese Medical Science (NO. 2017-021-SQ), Trial registration. China Clinical Trials Registry, chiCTR1800014277. The patients provided their written informed consent to participate in this study. Written informed consent was obtained from the individual(s) for the publication of any potentially identifiable images or data included in this article.

## Author contributions

JF conceived and designed this experiment. JS wrote the manuscript, revised it, and participated in the collection of cases and statistical analysis of the data. ZD drew diagrams, made statistical analysis of data, and revised the manuscript. YZ analyzed the data and revised the manuscript. YM, LC, ZW, CG, YL, and DG participated in data analysis, case collection, and manuscript writing. YH, LZ, MH, and JC performed fMRI on the patients. XH, XX, JT, and XY were involved in case collection and symptom assessment of patients. All authors contributed to the article and approved the submitted version.

## Funding

This research was supported by the National Natural Science Foundation of China (82174282 and 81774433), clinical efficacy and brain mechanism of transcutaneous auricular vagus nerve stimulation for patients with mild to moderate depression (QYSF-2020-02), China Academy of Chinese Medical Sciences Innovation Fund (CI2021A03301), and National Key Research and Development Program of China (2018YFC1705802).

## Conflict of interest

The authors declare that the research was conducted in the absence of any commercial or financial relationships that could be construed as a potential conflict of interest.

## Publisher's note

All claims expressed in this article are solely those of the authors and do not necessarily represent those of their affiliated organizations, or those of the publisher, the editors and the reviewers. Any product that may be evaluated in this article, or claim that may be made by its manufacturer, is not guaranteed or endorsed by the publisher.
